# Fever after bronchoscopy: serum procalcitonin enables early diagnosis of post-interventional bacterial infection

**DOI:** 10.1186/s12890-017-0508-1

**Published:** 2017-11-28

**Authors:** Klaus Hackner, Waltraud Riegler, Sabin Handzhiev, Rosemarie Bauer, Jan Veres, Manuela Speiser, Karin Meisinger, Peter Errhalt

**Affiliations:** 1 Department of Pneumonology, Krems University Hospital, Mitterweg 10, Krems, Austria; 2 Karl Landsteiner University of Health Science, Krems, Austria

## Abstract

**Background:**

The aim of this study was to differentiate unspecific and self-limiting fever after bronchoscopy from fever due to infection by using serum procalcitonin, C-reactive protein and neutrophil count. Furthermore, frequency of fever after bronchoscopy and procedures as possible risk factors were evaluated.

**Methods:**

Three hundred and fourteen consecutive patients were included. All bronchoscopies were performed using jet-ventilation and general anesthesia. Patients were analyzed according to interventions performed during bronchoscopy and laboratory results. Microbiological assessment was done in patients who developed fever to prove or rule out a bacterial infection.

**Results:**

Forty-four patients showed fever within 24 h following bronchoscopy (14%). A bacterial infection was proven in 11 patients with fever (3.5%). Procalcitonin, neutrophil count and C-reactive protein were significantly higher in patients with fever after bronchoscopy compared to non-fever patients. To predict bacterial infection in the receiver operating analysis, procalcitonin had the highest area under the curve (0.942; 95% confidence interval [CI], 0.768 to 1.000; *p* = <0.001), followed by neutrophil count (AUC, 0.804; 95% CI, 0.606 to 0.946; *p* = 0.005), whereas CRP levels where not statistically significant. Endoscopic airway recanalization was the only intervention that induced fever more frequently than all other interventions (OR 13.629).

**Conclusions:**

Fever is frequently seen after bronchoscopy and in some cases caused by bacterial infection. Procalcitonin might be useful to distinguish a bacterial infection from unspecific self-limiting fever. Airway recanalization is a procedure that seems to induce fever significantly more often than other bronchoscopic interventions.

## Background

Fever is a common phenomenon after a bronchoscopy but not always a sign of bacterial or viral infection. Since the beginning of flexible bronchoscopy in the early 1970s, a number of studies have reported transient fever within 24 h following the examination, estimating an incidence of 1.2–16% [1–5] for all bronchoscopic procedures.

The reason has not yet been fully discovered, however several authors described that especially bronchoalveolar lavage (BAL) may initiate an endogenous immune response. It is suggested that a systemic inflammatory response is induced, characterized by an increase in circulating cytokine levels released from alveolar macrophages such as tumor necrosis factor (TNF)-alpha, interleukin (IL)-1 beta, and IL-6 [6–10]. The effect of other bronchoscopic procedures (e.g. forceps biopsy or transbronchial needle aspiration) on the release of pyrogenic mediators has not yet been widely explored.

On the other side, endoscopic examinations may be the gateway through which bacteria invade the body. There have been reports of bacteremia and sepsis following bronchoscopy in patients with impaired immune system as well as healthy patients. The frequency ranges from 0.7 to 6.5% [11–16].

Consequently, fever after bronchoscopy is not always an endogenous response to the procedure with release of pyrogenic mediators, but may be caused by bacteremia. If patients have fever after bronchoscopy, a further post-interventional observation seems reasonable to assess the course and to start antibiotic treatment if needed. Blood cultures are the gold standard to diagnose systemic bacterial infection. However, laboratory parameters may help predicting a bacterial infection long before the microbiological workup is available.

Circulating proinflammatory mediators such as C-reactive protein (CRP) and more recently serum procalcitonin (PCT) have been suggested to be predictive for invasive bacterial infection [17, 18]. Especially PCT was largely investigated and seams to be helpful for the decision to start antibiotic therapy in respiratory infections. [19–21] Therefore, PCT becomes a more frequently used tool in clinical routine [22–25]. The stimulating impulses for high blood levels during bacteremia and sepsis are bacterial endotoxins (e.g. LPS) and cytokines (IL1-beta, TNF-alpha). [26] Within 6 h after systemic inflammation was induced, PCT levels begin to rise [27, 28].

We hypothesize that serum biomarkers are useful tools for ruling out or predicting bacteremia in fever after bronchoscopy, next to clinical signs and symptoms.

Therefore, we performed a study to evaluate the percentage of patients with fever due to infection after bronchoscopy and the role of biomarkers to early identify the patients demanding antibiotic treatment. Furthermore, as secondary outcomes we evaluated the prevalence of fever after bronchoscopy and procedures serving as risk factors (e.g. forceps or needle biopsy).

## Methods

### Patients and study design

This prospective observational study was performed at the pneumonology department of the university hospital in Krems, Austria.

Every patient referred for a bronchoscopy from May 2015 to January 2016 was included in the study after screening for inclusion and exclusion criteria. If patients underwent more than one bronchoscopy within the study period only the first procedure was taken into account.

Eligible subjects were at least 18 years. A reasonable indication for a diagnostic or therapeutic bronchoscopy was mandatory, as well as informed consent by the patients to undergo bronchoscopy and related data analysis. The local institutional review board and ethics commission approved the study.

Data collection included demographics, indication for bronchoscopy, procedures, microbiological results and laboratory findings pre- and post intervention. Patients with proven bacterial infection, elevated PCT levels (= ≥0.5 ng/mL) or ongoing antibiotic treatment before the bronchoscopy were excluded from the study.

### Bronchoscopy

Flexible, rigid or combined bronchoscopy was performed as an inpatient procedure and according to the guidelines of the European-respiratory-society [29]. All bronchoscopies were performed in jet-ventilation and the patients received general anesthesia. High frequency jet-ventilation (TwinStream**™**) was either performed in a supraglottic approach via larynx mask using a jet-adapter [30] or via rigid bronchoscope. For general anesthesia propofol, fentanyl or remifentanil were used. Rocuronium or mivacurium were used if muscle relaxation was necessary.

Bronchoscopy in local anesthesia is rarely performed at the study center (< 5% per year) and was therefore not included in the analysis.

During the procedure, continued monitoring of vital signs and oximetry was mandatory.

For flexible bronchoscopy, several Olympus video bronchoscopes (Olympus Corp., Lake Success, NY, USA) were used. After the inspection of the bronchial tree bronchoscopic procedures were performed at the discretion of the investigators, including BAL, forceps biopsy, transbronchial needle aspiration or other interventions (airway recanalization with cryoextraction, argon plasma coagulation and forceps; brushing; bronchial-fluid sample collection; treatment of hemoptysis; stent implantation; balloon-dilatation; lung volume reduction with valves).

For the BAL procedure, 200 mL of prewarmed 0.9% saline were instilled into the lobe of interest and then gently aspirated, according to the guidelines [31, 32].

At the study center, overnight observation after bronchoscopy is standard-of-care. Vital signs and oximetry were monitored for 6 h and a chest x-ray was performed if appropriate (e.g. after peripheral transbronchial biopsy). Adverse events were documented from the time of bronchoscopy until discharge.

### Measurement of temperature and laboratory parameters

Per protocol, 6 h and 24 h after bronchoscopy, the body temperature was measured by ear thermometer. Fever was defined as an elevated body temperature of ≥38 °C.

CRP levels, PCT levels, lymphocyte and neutrophil counts were obtained on the day prior to bronchoscopy and the day after bronchoscopy in all patients. PCT was measured using 100 μL of serum by an electrochemiluminescence immunoassay (ELECSYS® PCT; Brahms AG; Henningdorf, Germany) [17, 22, 33]. The assay has a analytical sensitivity ≤0.02 ng/mL and a functional sensitivity ≤0.06 ng/mL.

CRP was measured in heparin plasma using an enzyme immunoassay (CRPL3) with a detection limit of <0,5 g/dL in heparin plasma (Hitachi Instrument 917; Roche Diagnostics, Rotkreuz, Switzerland).

### Fever group

In patients who developed fever within 24 h after bronchoscopy we performed a microbiological workup, including stains and cultures for bacteria and fungi in blood and bronchial fluid. Positive bacterial cultures were counted as the number of colony-forming units per milliliter, and species identification and susceptibility tests were performed according to standard methods. Bacterial infection was diagnosed in cases with positive bacteriology results in the bronchial fluid and/or blood (i.e., a culture showing a single pathogenic microorganism above a minimum concentration of 10^4^ cfu/mL, excluding mouth flora).

### Statistical analysis

Discrete variables are expressed as absolute number (n) and percentage (%). Normally distributed data are shown as mean with standard deviation (SD), whereas non-normally distributed data are presented as median with interquartile range (IQR).

For all statistical analysis, SPSS software version 20.0.0 (IBM, Armonk, NY) was used. Differences were compared by Student’s *t* test, paired *t* test, Wilcoxon-Mann-Whitney test, chi-squared test, or Fisher’s exact test, as appropriate. Differences were considered statistically significant if *P* was <0.05. For correlation, spearman rho analysis was used when appropriate.

To evaluate the relationship among PCT, CRP, neutrophil count and proven bacterial infection, we constructed receiver operating characteristic (ROC) curves and determined the area under the curve (AUC). The AUC was considered to be clinically useful if it was ≥0.8 [34]. All statistical tests were two-tailed.

## Results

### Patient’s characteristics

Baseline characteristics are presented in Table 1. In total, 314 eligible patients received a bronchoscopy during the study period. The median age was 64 (interquartile range, 55 to 72 years). 114 bronchoscopies (36.3%) were performed in female patients. The indications for bronchoscopy are shown in Table 2.

**Table 1:** Baseline characteristics of the study patients

Patients characteristics	
Number of patients	314
Female, n (%)	114 (36.3)
Age at bronchoscopy, median (interquartile range)	64 (55–72)
Body temperature (°C), mean (SD):	
*Before bronchoscopy*	36.7 (0.37)
*After bronchoscopy, 6 h*	37.3 (0.73)
*After bronchoscopy, 24 h*	36.8 (0.38)
Fever after bronchoscopy (Temp ≥38 °C), n (%)	44 (14.0)

**Table 2:** Indications for 314 bronchoscopies

Underlying indication	Patients
Airway stenosis, suspected foreign body	5 (1.6)
Atelectasis	8 (2.5)
Bronchiectasis	2 (0.6)
Hemoptysis of unknown origin	12 (3.8)
Emphysema, lung volume reduction	1 (0.3)
Unclear lymphadenopathia	18 (5.8)
Suspected mucoid impaction	1 (0.3)
Suspected endobronchial polyp	2 (0.6)
Indication for airway recanalization	15 (4.8)
Indication for stent-implantation	2 (0.6)
Suspected interstitial lung disease	51 (16.3)
Suspected malignant tumor	148 (47.2)
Suspected pulmonary metastasis	10 (3.2)
Suspected mycobacteriosis other than tuberculosis	6 (1.9)
Suspected tuberculosis	16 (5.1)
Unclear radiologic infiltrative findings	17 (5.4)

The main examination technique was flexible (*n* = 280, 89.2%), followed by combined flexible and rigid (*n* = 29, 9.2%) and rigid-only bronchoscopy (*n* = 5, 1.6%). Bronchoscopic procedures were performed at the discretion of the physician performing the exam. Forceps biopsy was performed either peripheral guided by fluoroscopy or in the central airways under visual control. Transbronchial needle aspiration was guided by endobronchial ultra-sound. Suspicion of interstitial lung diseases was the main indication for BAL, most often combined with another procedure such as peripheral forceps biopsy or transbronchial needle aspiration.

We observed 5 pneumothoraces and two major bleedings, and no deaths related to bronchoscopy.

### Laboratory parameters and microbiological findings in post-bronchoscopic fever

Fever was observed in 44 patients after bronchoscopy (14%). Table 3 represents characteristics of laboratory results in the fever and non-fever cohort. Gender and age were almost equally distributed. Patients in the fever cohort had a slightly higher but not significant level of CRP, PCT and neutrophil count before bronchoscopy compared to the non-fever cohort. There is a consistent elevation of this parameters after bronchoscopy in both cohorts, nevertheless mean levels of CRP (11.03 mg/dL), neutrophil count (9.21 G/L) and PCT (0.78 ng/mL) were significantly higher in the fever group (p = <0.001, *p* = 0.001, *p* = <0.001). There was no difference in lymphocyte count in both cohorts before and after bronchoscopy. Mean PCT was elevated in both cohorts after bronchoscopy compared to the values before bronchoscopy, but the increase was significantly higher in the fever cohort (0.78 ng/mL; *p* = 0.029). In total, 13 patients (29.5%) in the fever cohort and 3 patients (0.1%) in the non-fever cohort showed values above 0,5 ng/mL which represents the upper limit for healthy persons.

**Table 3:** Comparison of laboratory results of patients with fever and without fever after bronchoscopy

	All (*n* = 314)	Fever (*n* = 44)	No fever (*n* = 270)	*P* value
Male gender, *n* (%)	200 (63.7)	30 (68.2)	170 (62.5)	n.s.
Age at bronchoscopy, median (interquartile range)	64 (55–72)	64 (53–73)	64 (56–72)	n.s.
Laboratory findings before bronchoscopy^a^, mean (SD)				
C-reactive protein (mg/dL)	2.58 (4.58)	5.04 (6.21)	2.18 (4.12)	n.s.
Absolute neutrophil count (G/L)	5.83 (2.63)	6.32 (3.66)	5.75 (2.42)	n.s.
Absolute lymphocyte count (G/L)	1.61 (0.71)	1.47 (0.59)	1.63 (0.72)	n.s.
Procalcitonin (ng/mL)	0.11 (0.37)	0.26 (0.21)	0.08 (0.22)	n.s.
Laboratory findings after bronchoscopy^b^, mean (SD)				
C-reactive protein (mg/dL)	4.12 (5.06)	11.03 (7.34)	3.06 (3.56)	<0.001
Absolute neutrophil count (G/L)	6.85 (3.21)	9.21 (4.41)	6.51 (2.84)	0.001
Absolute lymphocyte count (G/L)	1.72 (0.73)	1.46 (0.50)	1.76 (0.75)	n.s.
Procalcitonin (ng/mL)	0.19 (0.73)	0.78 (1.79)	0.11 (0.28)	0.029
Change of C-reactive protein (mg/dL), mean (SD)	+1.54 (3.39)	+5.82 (4.79)	+0.88 (2.54)	<0.001
Change of Procalcitonin (ng/mL), mean (SD)	+0.08 (0.38)	+0.47 (0.86)	+0.03 (0.23)	0.002
Change of neutrophil count (G/L), mean (SD)	+1.02 (3.17)	+2.88 (4.88)	+0.72 (2.68)	0.006
Change of lymphocyte count (G/L), mean (SD)	+0.10 (0.49)	−0.01 (0.33)	+0.12 (0.52)	n.s.
Elevated procalcitonin (≥0,5 ng/mL), n (%)	16 (5.1)	13 (29.5)	3 (0.1)	<0.001^†^

^a^The laboratory results were obtained on the day before bronchoscopy

^b^The laboratory results were obtained 12–24 h after the bronchoscopy

^†^Fisher’s exact test was applied

There was a moderate correlation between PCT and CRP levels in the fever cohort (Spearman rho = 0.472; *p* = <0.001) and between PCT and neutrophil count (Spearman rho = 0.513; p = <0.001). There was no correlation between PCT and lymphocyte count (*p* = 0.124).

To identify the main outcome parameter, microbiological workup was performed in the fever cohort. In 11 patients (3.5% of the study population) a bacterial infection was diagnosed by positive microbiological culture result in the bronchial fluid and/or blood. A detailed overview of these patients is given in Table 4. All of the patients with post interventional bacterial infection received empiric antibiotic or tuberculostatic treatment. Final culture reports and resistograms did not necessitate changes in the treatment in all patients.

**Table 4:** Documentation of post-interventional procalcitonin level and microbiological findings in patients with fever and proven bacterial infection after bronchoscopy

Noteable endoluminal findings	Microbiological findings	PCT, ng/ml
Purulent bronchial fluid	*Klebsiella pneumoniae* – BF	1.0
–	*E.coli* – BF, sputum	1.1
–	*Mycobacterium tuberculosis* – BF, pleural fluid	1.4
–	*Haemophilus influenzae* - BF	1.4
Purulent bronchial fluid	*P. aeruginosa* – BF, BC	1.3
Exophytic stenosis, poststenotic pneumonia	*S. pneumoniae* - BF, BC	11.1
purulent bronchial fluid	*E. coli* – BF, BC	4.4
Exophytic stenosis	*Haemophilus influenzae* - BF	1.8
Mucus plug, purulent bronchial fluid	*S. pneumoniae* - BF	0.2
Purulent bronchial fluid	*P. aeruginosa* – BF	0.3
Poststenotic purulent bronchial fluid	*S. pyogenes* - BF	2.6

*BF* Bronchial fluid

*BC* Blood culture

*PCT* Procalcitonin level (on the day after bronchoscopy)

In 33 patients with fever after bronchoscopy we could not find evidence of bacterial infection. Therefore, they were regarded to have nonspecific fever.

The fever patients were divided into those with proven bacterial infection and those with nonspecific fever and the laboratory findings were compared, as seen in Table 5. While there was a statistically significant difference in neutrophil counts (*p* = 0.014) and PCT (*p* = <0.001), CRP levels and lymphocyte counts showed no significant difference (determined by Wilcoxon-Mann-Whitney test).

**Table 5:** Laboratory parameters after bronchoscopy in patients having fever caused by proven bacterial infection and patients with unspecific fever

	Fever + proven bacterial infection (*n* = 11)	Unspecific fever (*n* = 33)	All (*n* = 44)	*p*-Value
CRP, mg/dL (SD)	14.76 (6.81)	10.03 (7.17)	11.03 (7.34)	n.s.
Neutrophils, G/L (SD)	13.11 (5.74)	7.93 (3.01)	9.21 (4.41)	0.014^a^
Lymphocytes, G/L (SD)	1.21 (0.60)	1.55 (0.49)	1.46 (0.50)	n.s.
Procalcitonin, ng/mL, (SD)	2.41 (3.10)	0.24 (0.27)	0.78 (1.79)	<0.001^a^

^a^Wilcoxon-Mann-Whitney test was applied

**Fig. 1:**
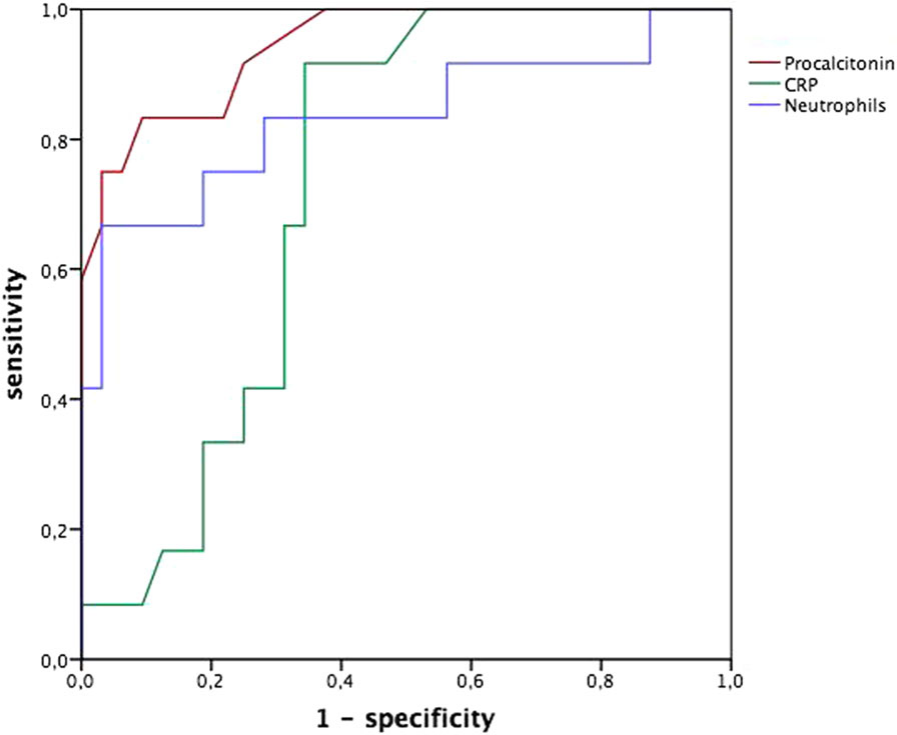
ROC analysis for prediction of bacterial infection causing fever after bronchoscopy: Procalcitonin level (AUC, 0.942; 95% confidence interval [CI], 0.768 to 1.000; *p* < 0.001), Absolute Neutrophil count (AUC, 0.804; 95% CI, 0.606 to 0.946; *p* = 0.005) and CRP levels (AUC, 0.729; 95% CI, 0.492 to 0.838; *p* = 0.094) are given

To determine the diagnostic value of laboratory parameters to predict a bacterial infection after bronchoscopy we performed ROC analysis (Fig. 1) in all patients with post-bronchoscopic fever (*n* = 44). PCT levels had the highest AUC (0.942; 95% CI, 0.768 to 1.000; *p* < 0.001), followed by neutrophil counts (AUC, 0.804; 95% CI, 0.606 to 0.946; *p* = 0.005). The AUC was not statistically significant for CRP levels (AUC, 0.729; 95% CI, 0.492 to 0.838; *p* = 0.094).

Sensitivity, specificity, Youden index and positive and negative likelihood ratio point estimates for neutrophil counts and PCT are presented in Table 6. At the threshold value of 10.0 G/L, neutrophil levels had a sensitivity of 72% and a specificity of 79%. PCT levels showed a specificity of 84% and a sensitivity of 81% at the threshold value of 0.5 ng/mL.

**Table 6:** Point estimates for absolute blood neutrophil count and procalcitonin to diagnose an underlying bacterial infection when fever after bronchoscopy occurs

Variables	Sensitivity	Specificity	LHR+	LHR-	J
Blood neutrophils, G/L					
5.0	90%	16%	1.07	0.63	0.06
10.0	72%	79%	3.43	0.35	0.51
12.0	63%	91%	7.00	0.41	0.54
Procalcitonin, ng/mL					
0.1	98%	60%	2.45	0.03	0.58
0.2	90%	73%	3.33	0.14	0.63
0.5	81%	84%	5.06	0.23	0.65
1.0	72%	97%	2.40	0.29	0.69

*LHR+* Positive likelihood ratio

*LHR*- Negative likelihood ratio

*J* Youden Index

### Distribution of bronchoscopic procedures in the two cohorts

The fever group was compared with the non-fever group to evaluate procedures as potential risk factor for post-interventional fever. Performing a forceps biopsy (odds ratio [OR] 0.513; 95% confidence interval [CI], 0.253 to 1.040: *p* = 0.061) or transbronchial needle aspiration alone (OR 1.588; 95% CI, 0.505 to 4.990: *p* = 0.425) showed no evidence of being an independent risk factor. Performing a BAL during bronchoscopy was no significant risk factor (OR 0.474; 95% CI, 0.162 to 1.390: *p* = 0.165), as well as performing a bronchoscopy without a BAL (OR 0.540; 95% CI, 0.262 to 1.116: *p* = 0.093).

There was no significant difference in the mean value of recovery fluid when BAL was performed (71.50 ml in the fever cohort and 76.60 ml in the non-fever cohort), or the mean number of specimen taken at forceps biopsy (4 in both cohorts) or transbronchial needle aspiration (3 in both cohorts).

Other procedures like brushing and bronchial-fluid sample collection, treatment of hemoptysis, stent implantation, balloon dilatation, airway inspection or endoscopic lung volume reduction with valves failed to show significance for post-bronchoscopic fever. Furthermore, various combinations of different procedures failed to show significantly more fever.

The only intervention associated with significantly higher frequency of fever was airway recanalization with forceps and argon plasma coagulation. From a total of 15 recanalization procedures, 10 patients developed fever (66.7%), with an OR of 13.629 (95% CI, 4.321 to 42.983: *p* = <0.001).

## Discussion

This is the first prospective study using inflammatory biomarkers to rule out or prove a bacterial infection when fever after bronchoscopy in general anesthesia occurs.

The prevalence of post-bronchoscopic fever was 14%. These finding is in line with previous studies, showing an incidence of fever after a bronchoscopy in 1.2–16% [1–4, 35].

The percentage of infectious fever after bronchoscopy was 3.5%.

Inflammatory biomarkers might be a useful approach to decide for an antibiotic therapy long before culture results would be available [17, 36]. The present findings illustrate that PCT levels are significantly higher in patients with a proven bacterial infection, when fever after bronchoscopy occurs. Interestingly, a rise of neutrophil granulocytes was another significant indicator for bacterial infection, proving an adequate immune response to the infection. The AUC for both laboratory tests showed very valuable results, therefore both of them should be considered in the decision making process.

A second outcome of this study was the investigation of different bronchoscopic procedures, serving as risk factor for post-bronchoscopic fever. Airway recanalization was the only intervention associated with significantly higher rates of fever in this study. We hypothesize that the destruction of tissue is causing a local and systemic inflammatory response. Furthermore, the disruption of epithelial integrity is a potential gateway for pathogens. Therefore, continuous monitoring after recanalization is advisable, with rapid counter-measures when signs of pulmonary bacterial infection or elevated biomarkers are noticed. In this subgroup (airway-recanalization) fever is frequent (66.7%) and early identification of patients requiring antibiotic therapy could be of clinical and economic benefit. The measurement of PCT the day after bronchoscopy could guide the decision of antibiotic treatment vs. discharge.

Other bronchoscopic interventions, such as forceps biopsy, transbronchial needle aspiration, or various combinations of these procedures did not cause fever. This finding is in line with several other studies [1, 3–5]. Despite previous reports [6–10], BAL did not provoke higher rates of fever.

Several limitations should be noted. First, we cannot provide information of complete microbiological workup for some patients without fever after bronchoscopy, because bronchial fluid collection was not performed in all of them.

Second, we did not implement multiplex polymerase-chain-reaction (PCR) technologies for the diagnosis of post-bronchoscopic infections, as they were not available during the study period at the study site. We suggest complete microbiological workup and the use of PCR technologies for further studies investigating this topic.

Third, we have evaluated clinical and laboratory parameters in regard to their diagnostic value on the day after bronchoscopy (not at the initiation of symptoms), and the kinetics of the biomarkers have not yet been analyzed.

Fourth, we did not evaluate procedure time or the impact of the used anesthetics.

Another limitation is the monocentric design. All included bronchoscopies were performed in jet-ventilation according to the clinics standard operating procedures. Although ventilation or anesthesia is unlikely to affect the outcome of this study, we recommend further multicenter approaches on this topic with inclusion of different ventilation, local anesthesia and evaluation of procedure time.

## Conclusions

In summary, our findings indicate that PCT and blood neutrophil counts seem to be useful tools to guide diagnostic and early therapeutic decisions for an underlying bacterial infection when patients develop fever after bronchoscopy,

Furthermore, endoscopic airway recanalization tends to be a risk factor for post-bronchoscopic fever.

## Abbreviations

AUC: Area under the curve
BAL: Bronchoalveolar lavage
CI: Confidence interval
CRP: C-reactive protein
IQR: Interquartile range
OR: Odds ratio
PCR: Polymerase chain reaction
PCT: Procalcitonin
ROC: Receiver operating characteristics
SD: Standard deviation
